# Spatial variations and controls of carbon use efficiency in China’s terrestrial ecosystems

**DOI:** 10.1038/s41598-019-56115-5

**Published:** 2019-12-20

**Authors:** Zhi Chen, Guirui Yu

**Affiliations:** 1 Synthesis Research Center of Chinese Ecosystem Research Network, and Key Laboratory of Ecosystem Network Observation and Modeling, Institute of Geographic Sciences and Natural Resources Research, Chinese Academy of Sciences, Beijing, 100101 China; 2 College of Resources and Environment, University of Chinese Academy of Sciences, Beijing, 100049 China

**Keywords:** Biogeography, Ecosystem ecology

## Abstract

Carbon use efficiency (CUE), one of the most important eco-physiological parameters, represents the capacity of plants to transform carbon into new biomass. Understanding the variations and controls of CUE is crucial for regional carbon assessment. Here, we used 15-years of continuous remote sensing data to examine the variations of CUE across broad geographic and climatic gradients in China. The results showed that the vegetation CUE was averaged to 0.54 ± 0.11 with minor interannual variation. However, the CUE greatly varied with geographic gradients and ecosystem types. Forests have a lower CUE than grasslands and croplands. Evergreen needleleaf forests have a higher CUE than other forest types. Climate factors (mean annual temperature (MAT), precipitation (MAP) and the index of water availability (IWA)) dominantly regulated the spatial variations of CUE. The CUE exhibited a linear decrease with enhanced MAT and MAP and a parabolic response to the IWA. Furthermore, the responses of CUE to environmental change varied with individual ecosystem type. In contrast, precipitation exerted strong control on CUE in grassland, while in forest and cropland, the CUE was mainly controlled by the available water. This study identifies the variations and response of CUE to environmental drivers in China, which will be valuable for the regional assessment of carbon cycling dynamics under future climate change.

## Introduction

Carbon use efficiency (CUE), the ratio between net primary productivity (NPP) and gross primary productivity (GPP), indicates how efficiently vegetation can convert carbon from the atmosphere into new plant materials^1,2^. CUE is thus a paramount ecological parameter determining not only vegetation carbon sink functioning but also the carbon cycling and turnover rate^3^. Quantifying the variations of CUE and its controls could promote a better understanding of carbon sequestration under climate change^3,4^.

The CUE has been assumed to be a constant in many previous studies^5,6^. For example, Waring *et al*.^7^ suggested that most global forests have an approximate CUE value of 0.47. Study in Canada’s temperate and boreal forests found that the CUE was stable across different species and stand ages^8^. However, the assumption of invariant CUE has been cast into doubt by increasing evidence from field data^2,9^. Through an integrated analysis, Delucia *et al*.^2^ noted that CUE substantially varied from 0.23 to 0.83 in diverse forest types. Across biomes, temperate forests have a CUE of 0.5, while tropical forests generally have a lower CUE of 0.2–0.4^10–12^.

Climate factors have been demonstrated to exert strong effects on the variations of CUE^13–15^. Driven by enhanced temperature and precipitation, the CUE was reported to vary with a decreasing trend in the eastern USA and globally^16–18^. Another analysis on global forests revealed that the CUE changed in a parabolic pattern along with temperature^15^. These studies have greatly enhanced our understanding of how CUE is affected by temperature and precipitation individually, while their interactive and combined effects have been generally less discussed. As a comprehensive parameter, the water availability index (IWA) has been shown to dominantly regulate patterns of carbon exchange rather than the individual variables of temperature and precipitation^19^. When considering hydrothermal conditions together, discrepant responses were found for CUE^18^. Moreover, the responses of CUE to environmental change are likely variable in diverse ecosystem types. Soil nutrients also play a key role in plant carbon allocation, which impacts CUE^20–23^. However, in most current studies exploring variations of CUE, the effects of water availability and soil nutrient factors have seldom been addressed.

Terrestrial ecosystems in China play a remarkable role in balancing atmospheric carbon dioxide, with high photosynthetic capacity of up to 7.78 Pg C y^−1 ^^24–26^. How efficiently these ecosystems can convert photosynthates into plant and soil storage greatly determines regional carbon sequestration and their feedback to climate change^25,27^. Studies have investigated the CUE in China. For example, the CUE was estimated to be 0.34 in a primary tropical seasonal rain forest^28^. The root CUE decreased with stand age in a Chinese fir plantation^29^. However, most of these studies on CUE mainly discussed individual ecosystem types and focused on small plots at the stand or site level. The spatial variations of CUE have rarely been explored at the regional scale, and the impacts of climatic and soil factors on the CUE have not been well quantified. In particular, the roles of water availability and soil nutrients have not been taken into sufficient consideration.

Moderate Resolution Imaging Spectroradiometer (MODIS) products pertaining to GPP and NPP have been widely used from regional to global scales to calculate CUE^16,17,30,31^. These products have been validated to have the capability to capture spatial and temporal patterns of GPP and NPP across various biomes and climate zones^16,32^. Additionally, an upgraded global soil nutrient dataset composed of data on the contents of multiple nutrients has become available^33^. These regionally continuous data provide a unique approach to examine the spatial patterns of CUE and the relationships of CUE with climate and soil factors.

Here in the current study, we used 15 years of continuous remote sensing CUE data integrated with vegetation, climate and soil data to (1) explore the spatial variations of CUE across China; (2) identify the trends in CUE across climatic and soil gradients; and (3) determine the variations and responses of CUE to climatic and soil gradients among different ecosystem types. These findings could advance our knowledge of changes in regional carbon balance in response to climate change.

## Results

### Spatial distribution of CUE in China

**Figure 1:**
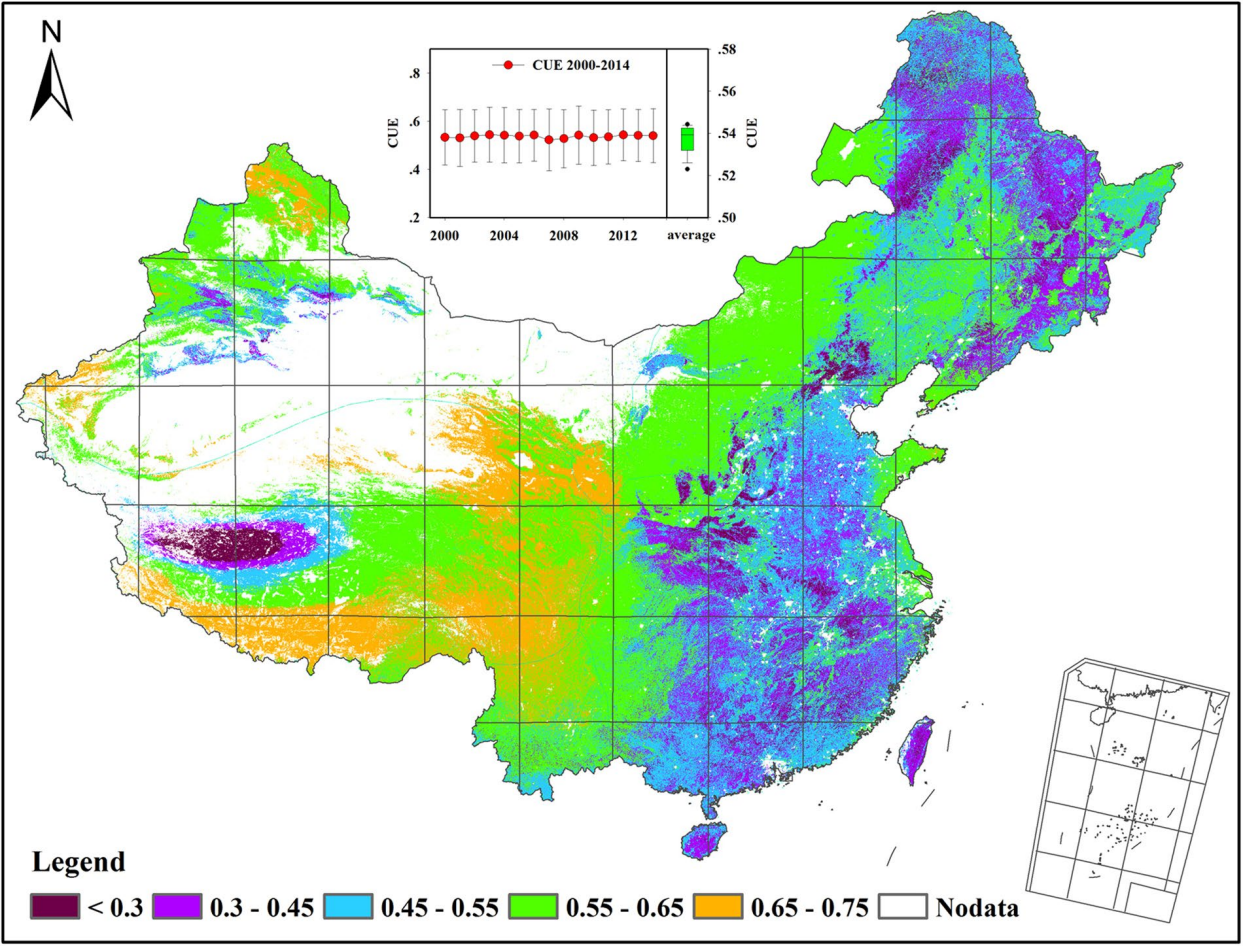
The spatial distribution of mean carbon use efficiency (CUE) across China’s ecosystems.

From 2000 to 2014, the vegetation CUE was averaged to be 0.537 ± 0.114 across the whole China region (Fig. 1). During the past 15 years, the mean CUE showed slight fluctuations, varing from 0.544 ± 0.114 in 2003 to 0.523 ± 0.128 in 2007 (Fig. 1). This minor interannual variation in CUE indicates that CUE has high annual stability.

The CUE showed large spatial variations across China that were characteristic of low in low-altitude and humid areas and high in high-altitude and dry areas (Fig. 1). The eastern edge of the Tibetan Plateau and Qilian Mountains showed the largest vegetation CUE, with a mean annual CUE of up to 0.65–0.75. The central Tibetan Plateau, Yunnan-Guizhou Plateau, Loess Plateau, eastern part of the Inner Mongolian Plateau and Junggar Basin had CUE values larger than 0.55. The vegetation CUE was relatively low in the Northeast Plain, North China Plain and southeastern part of China, being below 0.5.

**Figure 2:**
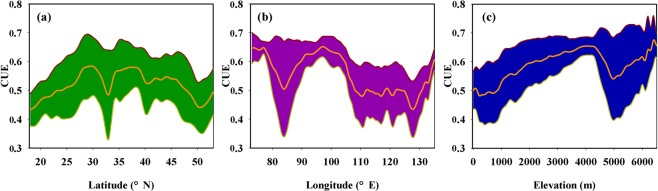
The geographic pattern of mean carbon use efficiency (CUE) along with latitude (**a**), longitude (**b**) and elevation **(c**) in China. The thin line represents the average CUE value at intervals of 1° latitude and longitude, and 100 m altitude, respectively; the colored band represents the ±1 SD (standard deviation) range.

Geographically, the spatial variations of CUE exhibited complex horizontal zonality with latitude and longitude (Fig. 2). Along the latitudinal gradient, the CUE increased from 0.43 to 0.58 around 30°N and then dropped to 0.48 around 33°N. From 33° to 37°N, the CUE increased from 0.48 to 0.58 and then dropped to 0.53 around 40°N. Between 40° and 45°N, the CUE stabilized around 0.54 and then decreased to 0.44 around 50°N (Fig. 2a). Along the longitudinal gradient, the CUE decreased from 0.64 to 0.50 around 85°E and then increased to 0.65 around 97°E. Above 97°E, the CUE decreased to 0.48 around 110°E and then stabilized between 110°E and 125°E. From 128° to 135°E, the CUE increased from 0.43 to 0.61 (Fig. 2b). Overall, the CUE followed an initial increasing and subsequent decreasing trend with latitude, with the largest CUE appeared in the mid-latitude areas. In term of longitude, the CUE decreased from the west to the east.

The spatial variations of CUE exhibited clear vertical zonality with altitude (Fig. 2c). From sea level to 4000 m, the CUE increased from 0.48 to 0.65 and then dropped to 0.54 around 5000 m. Above 5000 m, the CUE continued to increase to 0.65 around 6500 m. In comparison to that in low-latitude areas, the CUE increased at a faster rate in high-latitude areas (Fig. 2c).

### Variations of CUE in different ecosystem types

Across different biomes, the GPP and NPP decreased from tropical to subtropical, temperate and alpine zones, while the CUE showed the opposite trend (Table 1). A large CUE value was observed for alpine vegetation (0.58), which indicates a low productivity but high CUE the alpine vegetation does have. In addition, within similar temperate zone, the temperate desert had a larger CUE than the temperate grassland and temperate forest. This difference in CUE among temperate forest, grassland and desert demonstrates that vegetation type has an obvious impact on the CUE.

**Table 1:** 2000–2014 mean annual GPP, NPP and CUE for different biomes.

Biomes	Mean annual GPP (g C m^−2^ yr^−1^)	Mean annual NPP (g C m^−2^ yr^−1^)	Mean annual CUE
Tropical forest	1558.84 ± 793.03	819.71 ± 409.3	0.547 ± 0.097
Subtropical forest	1183.35 ± 499.16	596.93 ± 252.94	0.526 ± 0.110
Warm temperate forest	715.31 ± 207.56	354.51 ± 114.65	0.503 ± 0.103
Temperate forest	790.88 ± 156.69	367.99 ± 87.19	0.469 ± 0.093
Temperate grassland	374.64 ± 154.17	203.81 ± 75.76	0.561 ± 0.075
Temperate desert	248.23 ± 187.30	143.48 ± 96.07	0.598 ± 0.071
Cold temperate forest	712.01 ± 95.08	319.37 ± 83.78	0.443 ± 0.091
Alpine vegetation	128.52 ± 193.15	79.98 ± 102.89	0.581 ± 0.127

**Figure 3:**
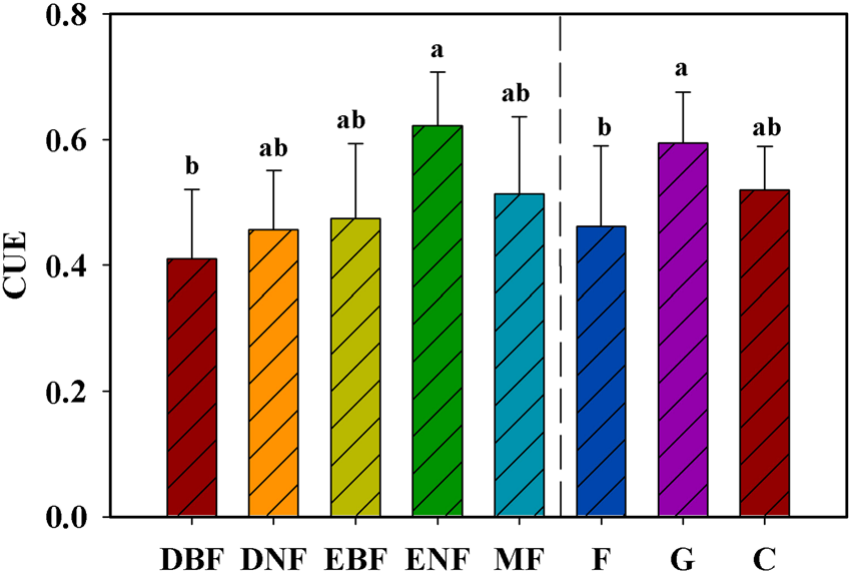
The mean annual CUE of different ecosystem types. DBF, DNF, EBF, ENF, and MF represent deciduous broadleaf forests, deciduous needleleaf forests, evergreen broadleaf forests, evergreen needleleaf forests and broadleaf and needleleaf mixed forests, respectively; F represents forests including DBF, DNF, EBF, ENF, and MF; C represents croplands; and G represents grasslands. The column and error bars represent the mean ± standard deviation. Different letters indicate significant differences at the level of α = 0.05.

The analysis of variance (ANOVA) indicated that there was a significant difference in CUE among ecosystem types (*P* < 0.05) (Fig. 3). The highest mean CUE (0.59) was observed in grassland, and the lowest mean CUE (0.46) was identified in forest. Further analysis showed that evergreen needleleaf forests had the largest CUE, which was significantly higher than that of deciduous broadleaf forests. There were comparable CUE values among deciduous needleleaf forests, evergreen broadleaf forests, and broadleaf and needleleaf mixed forests. Needleleaf forests had a higher CUE than broadleaved forests, and evergreen forests had a higher CUE than deciduous forests (Fig. 3). However, this difference was not significant at the significance level of 0.05.

### Correlations between CUE and environmental factors

The effects of climatic and soil factors on the spatial variations of CUE were examined by correlation analysis (Table 2). The results showed that the CUE was negatively related to the MAT, MAP, IWA and soil clay percentage, while it was positively related to the soil sand percentage, SOC, pH, and CEC. Different ecosystems showed divergent relations with the climate and soil variables. Although all factors exhibited significant relationships with CUE (*P* < 0.05), climate had a stronger effect on CUE than soil variables, and soil SOC, Clay and pH exhibited a stronger effect than soil Sand and CEC. We further used linear and nonlinear regressions to analyze the spatial responses of CUE to the influencing climatic variables (MAT, MAP and IWA) and soil factors (Clay, SOC and pH).

**Table 2:** Correlation coefficients between CUE and climatic and soil factors in China.

	MAT ( °C)	MAP (mm)	IWA (mm/mm)	Sand (%wt.)	Clay (%wt.)	SOC (%wt.)	pH (−log(H^+^))	CEC (cmol/kg)
All	−0.206	−0.274	−0.350	0.126	−0.220	0.054	0.105	0.057
	<0.001	<0.001	<0.001	<0.001	<0.001	<0.001	<0.001	<0.001
Forest	0.123	0.018	−0.102	−0.034	0.013	0.039	−0.069	−0.027
	<0.001	<0.001	<0.001	<0.001	<0.001	<0.001	<0.001	<0.001
Grassland	−0.032	0.239	0.171	0.044	−0.080	0.081	−0.056	0.004
	<0.001	<0.001	<0.001	<0.001	<0.001	<0.001	<0.001	<0.001
Cropland	−0.114	−0.153	−0.326	0.059	−0.024	0.008	−0.052	−0.028
	<0.001	<0.001	<0.001	<0.001	<0.001	<0.001	<0.001	<0.001

MAT, mean annual temperature; MAP, mean annual precipitation; IWA, index of water availability; SOC, soil organic content; and CEC, cationic exchange capacity.

**Figure 4:**
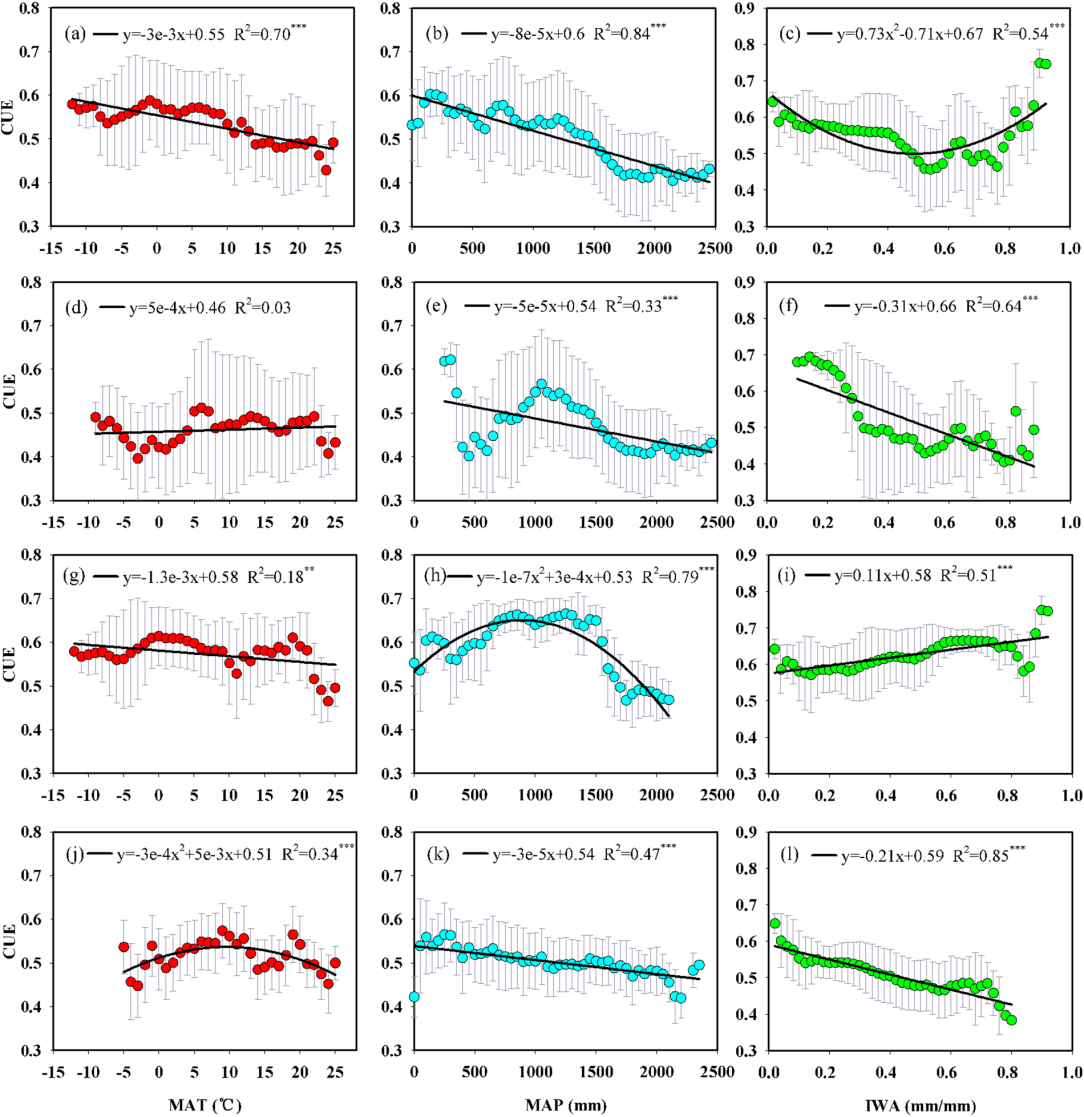
Relationships between CUE and climatic factors across the whole region (**a–c**) and in individual ecosystem types (forest: **d–f**; grassland: **g–i**; cropland: **j–l**). MAT, mean annual temperature; MAP, mean annual precipitation; IWA, index of water availability. The circles and error bars represent the mean ± standard deviation at intervals of 1 °C MAT, 50 mm MAP and 0.02 mm/mm IWA, respectively. *, **, and *** indicate that the regression equation was significant at the 0.05, 0.01 and 0.001 levels, respectively.

With an increase in MAT, the CUE showed a significant linear decreasing trend across the whole region (Fig. 4a) and in grasslands (Fig. 4g). However, the CUE showed a quadratic response to MAT in cropland (Fig. 4j) and exhibited no significant correlation with MAT in forest (Fig. 4d). Along with precipitation, the CUE represented a significant linear decreasing trend across the whole region (Fig. 4b), in forest (Fig. 4e) and cropland (Fig. 4k), while it showed a quadratic response to precipitation in grassland, with the largest CUE at 1000 mm (Fig. 4h). The variations of CUE along with the IWA exhibited much higher divergence than that with MAT and MAP. An initial decreasing and subsequent increasing trend in the CUE was found along with the IWA across the whole region (Fig. 4c). Similar relationships were found between the CUE and another water index (potential evapotranspiration ratio, PET/P) (Fig. S1). In forest (Fig. 4f) and cropland (Fig. 4l), the CUE decreased with the IWA, while the CUE increased with the IWA in grassland (Fig. 4i). In contrast, precipitation exerts strong control on the CUE in grassland, while in forest and cropland, the CUE was mainly controlled by the available water.

**Figure 5:**
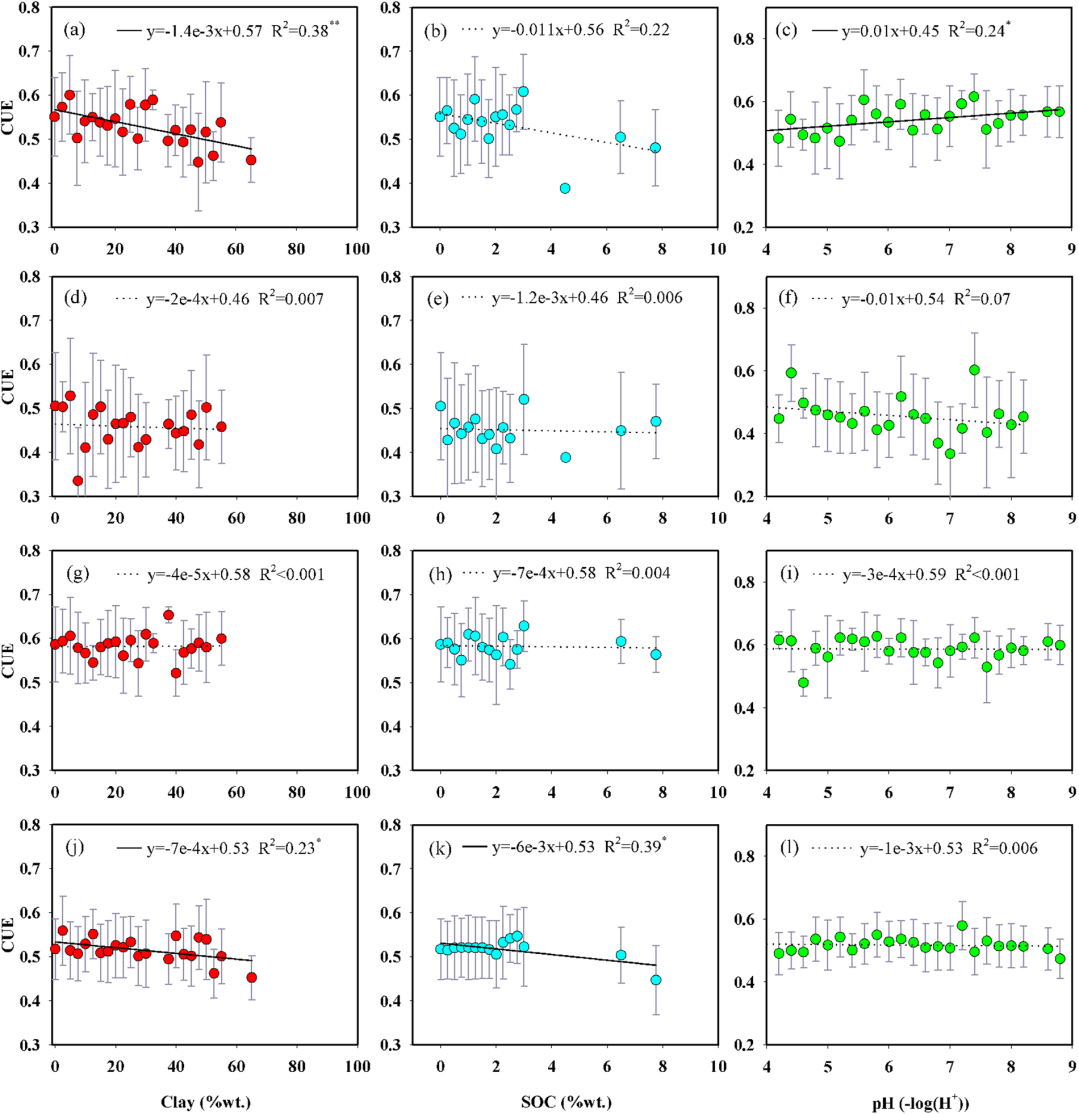
Relationships between CUE and soil factors across the whole region (**a–c**) and in individual ecosystem types (forest: **d–f**; grassland: **g–i**; cropland: **j–l**). SOC, soil organic content. The circles and error bars represent the mean ± standard deviation at intervals of 2.5% Clay, 0.25% SOC and 0.2 pH, respectively. *, **, and *** indicate that the regression equation was significant at the 0.05, 0.01 and 0.001 levels, respectively. The dotted line indicates that the regression equation was not significant at the 0.05 level.

The soil clay percentage and SOC exerted negative effects on the CUE across the whole region (Fig. 5a,b) and in cropland (Fig. 5j,k), while no evident relationship was found in forest (Fig. 5e) and grassland (Fig. 5h). The CUE linearly increased with pH across the whole region (Fig. 5c), while soil pH exerted no apparent effect on the CUE in forest (Fig. 5f), grassland (Fig. 5i) and cropland (Fig. 5l).

## Discussion

### Variations of CUE in china’s ecosystems

Our integrated analysis of the 15 years of mean annual CUE data showed that China’s terrestrial ecosystems have an average CUE of 0.54 ± 0.11, suggesting that, on average 54% of photosynthetic production would be stored in plants as new materials in ecosystems. The global average CUE was estimated to be 0.52 by remote sensing data^16^ and 0.45 by process-based models^18^, respectively. The CUE across China was higher than the average level of global ecosystems, which implies a relatively high level of carbon transfer efficiency in China’s terrestrial ecosystems.

The CUE of Chinese ecosystems presented minor interannual variation, while large spatial variations were associated with the geographic distribution. Clear horizontal and vertical zonality of CUE along with latitude, longitude and altitude was found across China. With increasing latitude, the CUE followed an initial increasing and subsequent decreasing trend. The greatest CUE appeared in the mid-latitude areas, which was consistent with the reported latitudinal pattern of global CUE and carbon sequestration^13,14,16^. This parabolic pattern most likely occurs because of the high cost of respiration associated with warm conditions at low-latitudes^34,35^, and substantial loss of C during the dormant season but low productivity input under the restraint of low temperatures in high-latitude areas^36,37^.

In terms of the longitudinal pattern, the CUE generally decreased from the west to the east. This pattern was closely coupled with the altitudinal variations of CUE. From the west to the east, the topography transfers from the plateau to the plain across China. Our results presented a corresponding rise in CUE as the altitude increased. At the regional and global scales, Kwon *et al*.^17^ and Zhang *et al*.^16^ similarly revealed that CUE increased with increasing elevation. Latitude is a combined factor surrogate for changes in temperature, precipitation and radiation. Previous studies have indicated that plants in cold environments expend less energy on maintaining living organisms relative to those in warm environments^38,39^. As an example, Zach *et al*.^40^ indicated that the fraction of stem growth respiration from the total respiration decreased from 14% at 1,050 m to 10% at 3,050 m. In addition, NPP was also reported to have higher resilience to extreme environments than GPP, which was expected to lead to a high level of CUE at high latitudes^16^.

### Effects of ecosystem types on CUE

The CUE is assumed to be a constant and was widely used to quantify plant respiration in early carbon cycle models, such as the CASA and BGC models^41,42^. However, increasing evidence from field data demonstrates that CUE substantially varies with ecosystem type, stand structure and forest age^2,10,43^. Our results showed that the CUE varied significantly in association with vegetation type, which provides strong support for a variable CUE. Forests were found to have a lower CUE than grasslands and croplands. This result is confirmed by previous studies suggesting that grasses and crops have a higher CUE than forests^16,44^. For grasses and crops, the CUE values are reported to be nearly 30% higher than those for forests^44^. In comparison to natural vegetation, crops likely have a high CUE value due to their suitable climatic conditions and soil nutrients supply^45,46^.

Significant differences in CUE were also found among forest types in this analysis. The highest mean CUE was found in evergreen needleleaf forests, with a value of 0.62 ± 0.08. This efficiency was higher than the average level of global evergreen needleleaf forests (0.45–0.58)^16,47^, which suggests that evergreen needleleaf forests in China have high carbon transfer efficiency. However, this is inconsistent with findings of analysis conducted by Zhang *et al*.^16^ and Collalti *et al*.^47^, who found low CUE in evergreen needleleaf forests at the global scale. This discrepancy can most likely be attributed to the difference in forest management and stand age. Most evergreen needleleaf forests in China are managed by humans, and, more importantly, they are currently of young age and have been demonstrated to have high carbon sequestration capacity and CUE^48,49^.

### Effects of climatic and soil factors on CUE

Numerous studies have indicated that variations of climatic factors has great effects on NPP and GPP at regional and global scales^25,50–54^. As the fraction of NPP to GPP, the CUE is expected to vary substantially with environmental factors. Previous study by Zhang *et al*.^16^ showed that the CUE decreased between −20 °C and −10 °C, and increased between −10 °C and 20 °C along with the arising temperature at the global scale. Piao *et al*.^15^ revealed that the CUE varied in a parabolic pattern across global forests, with the highest CUE observed around 11 °C. Currently, more consistent decreasing trends of CUE have been found in studies utilizing multiple processed-based models and remote sensing data^17,18^. Our results showed that with increasing temperature, the CUE represented a significant linear decreasing trend in the China region. Although a rising trend appeared between 0 °C and 10 °C, the overall decreasing trend did not change. This may be attributed to the fact that in a warmer environment, vegetation has relatively lower productivity storing efficiency due to the enhanced respiratory cost associated with increasing temperature^11,41,55^.

Precipitation is another factor strongly influencing CUE. The CUE linearly decreased with enhanced precipitation across China. This trend was in agreement with the analysis at the global scale that found the CUE declined along with arising precipitation when it was less than 2000 mm^16,18^. The decrease in CUE with precipitation may be the combined result of multiple factors, including reduced radiation, enhanced nutrient leaching, a shortage of soil oxygen, slowed organic matter decomposition and the nutrient supply^56^.

In addition to temperature and precipitation alone, the combined effects of heat and water conditions are expected to exert great effects on carbon cycles. Reichstein *et al*.^19^ indicated that spatial variations of carbon components were primarily controlled by the IWA across European forests. This analysis showed that the CUE greatly varied with the IWA across China and presented an initial decreasing and subsequent increasing trend along with the IWA. With an increase in the IWA, the CUE decreased when the IWA was below a threshold of 0.5. This result implies that the fraction of respiration cost in total assimilation increases when the IWA is lower than 0.5. In previous study, productivity has been found to have no evident trend, while respiration positively increased, therefore resulting in a decreasing pattern of CUE with the increase of the IWA^13^. This trend was clearly shown in forest and cropland in this analysis. In addition, our results also found that the responses of CUE to environmental change varied with individual ecosystem type. In contrast, precipitation exerted strong control on CUE in grassland, while in forest and cropland, the CUE was mainly controlled by the available water. This implies that precipitation is a strong limiting factor of CUE in grassland, while in forest and cropland, CUE relies more strongly on the combined hydrothermal conditions.

In comparison to climate factors, soil conditions played minor effects on the variations of CUE in China. This result provides no support to the global analysis showing that soil nutrient availability regulates the global CUE^21–23^. This discrepancy may be associated with the different classification of soil variables and limited variation ranges in China^21,23^. However, we found a decreasing trend in CUE with the soil clay percentage and an increasing trend with the soil pH value. This pattern was largely in agreement with the transition of ecosystem type from forests (high soil clay percentage and low pH value) to grasslands (low soil clay percentage and high pH value).

This study analyzed the regional characteristics of CUE and underlying regulating factor, which can be useful for carbon budget assessment and ecosystem management. It is worth noting that, there were several uncertainties stem from the MOD17 GPP products and statistical processes. The underlying errors in the interpolated climate data and the LAI/fPAR algorithm likely caused potential errors in MOD17 GPP products. Besides, the errors in land cover classification affects the regional statistics of CUE. Compared to the single-type pixels for forest, grassland and cropland, higher uncertainties of CUE were shown in the transition zones of grassland and barren lands, and in cropland/natural vegetation mosaics. A high-precision land cover product over China is needed to further improve the regional assessment of CUE in the future.

## Conclusion

This study examined variations of CUE across broad geographic, vegetation, and climatic gradients in China’s ecosystems. The results provide strong support for a variable CUE. Geographically, the CUE exhibited a clear horizontal zonality along with latitude and longitude and a clear increasing pattern along with altitude. Ecosystem types and climatic conditions exert strong effects on CUE across China. Forests have a lower CUE than grasslands and croplands. Evergreen needleleaf forests have a higher CUE than other forest types. The CUE varied in a linear decreasing trend with increasing MAT and MAP, while an initial decreasing and subsequent increasing trend along with the IWA. Moreover, the responses of CUE to environmental change varied with individual ecosystem type. In contrast, precipitation exerted strong control on the CUE in grassland, while in forest and cropland, the CUE was mainly controlled by the available water. This study advances our knowledge of the variations and responses of CUE to environmental drivers in China, which could be helpful to understand the dynamics of carbon cycling processes in response to future climate change at the regional scale.

## Material and Method

### Data sources

#### GPP and NPP data

GPP data derived from the Moderate Resolution Imaging Spectroradiometer remote sensing product (MOD17A3). GPP from MOD17A3 was calculated by the concept of radiation conversion efficiency, and expressed as

$${\rm{GPP}}={\rm{\varepsilon }}\times {\rm{PAR}}\times {\rm{FPAR}}$$

where ε is the radiation use conversion efficiency of the vegetation; PAR is the photosynthetically active radiation; and FPAR is the fraction of incident PAR that was absorbed by the surface, respectively. PAR was determined from meteorological observations, and was estimated as 45% of the incident shortwave radiation. FPAR was obtained from the remote sensing product MOD15. The ε was the reduced ε_max_ confined by low temperature (*f*T_min_) and water stress (*f*VPD), where ε_max_ was obtained from the biome properties look-up table (BPLUT).

NPP is the remaining GPP after maintenance and growth respiration (R_m_, R_g_), and expressed as

$${\rm{NPP}}={\rm{GPP}}-{{\rm{R}}}_{{\rm{m}}}-{{\rm{R}}}_{{\rm{g}}}$$

To model respiration, the R_m_ and R_g_ are inferred from the allometric relationships that link the biomass and annual growth of plant tissues to the satellite-derived leaf area index (LAI). Leaf mass was first estimated as the ratio of LAI to the specific leaf area (SLA), where LAI was obtained from the MOD15 and SLA was obtained from the BPLUT. Root biomass was subsequently calculated as the product of leaf mass and the root/leaf ratio, and livewood mass was obtained from the product of the annual maximum leaf mass and livewood/leaf ratio, where both the root/leaf ratio and livewood/leaf ratio were obtained from the BPLUT. The R_m_ of leaf, root and livewood was calculated on a daily basis based on the maintenance respiration rate per unit of leaf, root, and livewood biomass, respectively. The annual leaf R_g_ was first inferred from the relationships that link the annual maximum leaf mass and leaf base growth respiration. The annual R_g_ of root, livewood and deadwood were then calculated with its ratio to leaf R_g_ that were obtained from the BPLUT.

The annual GPP and NPP data (MOD17A3) at 1 × 1 km resolution from 2000 to 2014 in TIF format that were used in this study were downloaded from the Numerical Terradynamic Simulation Group (NTSG) at the University of Montana (http://www.ntsg.umt.edu/). This dataset has been widely used to analyze the temporal and spatial patterns of biomass, productivity and carbon cycles at the regional and global scales^30,31,57^. We coordination projection converted, clipped and resampled the downloaded grid data to obtain the annual GPP and NPP dataset for the China region. To reduce the impacts of anomalies, the annual GPP and NPP values from 2000 to 2014 outside the range of ±3 times standard deviation were eliminated. Finally, the 15-year average (2000–2014) annual GPP and NPP values were calculated for each pixel. The GPP product was validated using the eddy flux towers observation dataset. The dataset consists of 153 annual GPP data from 49 eddy flux towers over China, and the results demonstrated that the MODIS GPP was in good agreement with the site observed GPP data (Fig. S2 and Table S1).

#### Land cover and topographic data

The 1 × 1 km resolution land cover data were retrieved from the MOD12Q1 product (https://modis.gsfc.nasa.gov/data/dataprod/mod12.php). According to the land cover classification schemes defined by the International Geosphere-Biosphere Project (IGBP), the global land cover was classified into 17 types, including 11 natural vegetation classes, 3 human-altered classes, and 3 non-vegetated classes. The land cover categories used in this study include evergreen needleleaf forest, evergreen broadleaf forest, deciduous needleleaf forest, deciduous broadleaf forest, mixed forest, grassland, and cropland. The MODIS land cover product over China has been validated by comparison with data from the National Land Use/cover Database of China (NLUD-C). The results showed that the overall accuracy of MODIS land cover product was up to 66.42% at the pixel scales which have high producer and user accuracy for cropland, grass and forests^58^.

The Global Land One-kilometer Base Elevation (GLOBE) data derived from the National Oceanic and Atmospheric Administration (https://www.ngdc.noaa.gov/mgg/topo/gltiles.html) were used in this study. The downloaded elevation data were coordination projection converted, clipped and resampled to match the GPP and NPP data.

#### Climatic data

Climatic factors including annual temperature and precipitation (MAT, MAP) from 2000 to 2014 at the 1 km resolution were obtained from the interpolated temperature and precipitation dataset (http://www.cnern.org.cn/data/). The dataset was produced based on the observed daily precipitation and mean temperature data collected from 753 and 345 ground meteorological stations from the Daily Global Historical Climatology Network-Daily (GHCN-D) and the National Meteorological Information Center (NMIC) of the China Meteorological Administration. The temperature and precipitation data were standardized and interpolated to the grid with 1 km resolution using the ANUSPLIN software^59^.

The index of water availability (IWA) was calculated as the ratio of annual actual evapotranspiration (AET) to potential evapotranspiration (PET)^19^. We downloaded the annual AET and PET data (MODIS16A3) from 2000 to 2014 at 1 × 1 km resolution in TIF format from the Numerical Terradynamic Simulation Group (NTSG) at the University of Montana (http://www.ntsg.umt.edu/). To match the GPP and NPP data, the downloaded AET and PET data were coordination projection converted, clipped and resampled. The IWA of each year was first calculated and then the 15-year average (2000–2014) annual IWA was estimated for each pixel in the China region.

#### Soil data

Soil data were obtained from the regridded Harmonized World Soil Database (version 1.2) (https://daac.ornl.gov/cgi-bin/dsviewer.pl?ds_id=1247) produced by the Food and Agriculture Organization of the United Nations (FAO) and the International Institute for Applied Systems Analysis (IIASA). The soil physical and chemical characteristics, including the percentages of sand (Sand) and clay (Clay), soil pH (pH), soil organic content (SOC) and cationic exchange capacity (CEC) of the topsoil at the depth of 0–30 cm were extracted in this study.

#### Data analysis

The average CUE for 15 years (2000–2014) was calculated and used to analyze the spatial variations of CUE. Further analysis was performed to examine the difference in CUE among different biomes. To examine the geographic pattern, the mean annual CUE was plotted against latitude, longitude and elevation respectively. The One-way analysis of variance (ANOVA) was conducted to determine how CUE varies with different types of forests and ecosystems at the significance level of α = 0.05. The normal distribution and homogenous variance of data were first examined, and a Least Significant Difference (LSD) post hoc test was followed to identify the exact differences. The Pearson correlation coefficients between CUE and the climatic variables (MAT, MAP, and IWA) and soil factors (Sand, Clay, SOC, pH and CEC) were calculated to evaluate the sensitivity of vegetation CUE to the climatic and soil variables. A high R-value represents a strong relationship and vice versa. In addition, the relationships of CUE and climatic and soil variables were examined for each individual ecosystem type. Linear and nonlinear regressions were further used to analyze correlations of CUE with its influencing factors. All data analyses were performed using SPSS 20.0 and MATLAB R2014a software.

## Supplementary information

Dataset 1
