# Supplementary material for: Spatial variations and controls of carbon use efficiency in China’s terrestrial ecosystems
Source: Sci Rep. 2019 Dec 20;9:19516. doi: 10.1038/s41598-019-56115-5 (PMC6925132; doi:10.1038/s41598-019-56115-5)
Supplement: Supplementary file 1 — Dataset 1 [file 41598_2019_56115_MOESM1_ESM.docx]

**Spatial variations and controls of carbon use efficiency in China’s terrestrial ecosystems**

Zhi Chen^1,2*^, Guirui Yu^1,2*^

^1^Synthesis Research Center of Chinese Ecosystem Research Network, and Key Laboratory of Ecosystem Network Observation and Modeling, Institute of Geographic Sciences and Natural Resources Research, Chinese Academy of Sciences, Beijing 100101, China.

^2^College of Resources and Environment, University of Chinese Academy of Sciences, Beijing 100049, China

Correspondence should be addressed to Z.C. ([chenz@igsnrr.ac.cn](mailto:chenz@igsnrr.ac.cn)) or G.Y. (yugr@igsnrr.ac.cn).

**Figure S1** Relationships between CUE and potential evapotranspiration ratio (PET/P) across the whole region (a) and in individual ecosystem types (forest: b; grassland: c; cropland: d).

The circles and error bars represent the mean ± standard deviation at intervals of 0.2 mm/mm in forest and 1 mm/mm across the whole region and in grassland and cropland, respectively. *, **, and *** indicate that the regression equation was significant at the 0.05, 0.01 and 0.001 levels, respectively.





**Figure S2** The relationships between annual GPP estimates from the flux tower and the MODIS GPP product over China.





**Table S1** Sites characteristics of this study

| Site number | Sites  name | Latitude (°N) | Longitude (°E) | Altitude (m) | Ecosystem types | GPP (g C m^-2^yr^-1^) | References |
| --- | --- | --- | --- | --- | --- | --- | --- |
| 1 | Xishuangbanna | 21.95 | 101.20 | 750 | Forest | 2342.67 | Zhang et al. (2010a) |
| 2 | Dongguan Garden | 22.97 | 113.74 | 40 | Urban Garden | 1482 | Sun et al. (2012) |
| 3 | Dinghushan | 23.17 | 112.53 | 300 | Forest | 1367.26 | Yu et al. (2013) |
| 4 | Yuanjiang | 23.47 | 102.18 | 553 | Grassland | 684 | Fei et al. (2017) |
| 5 | Ailaoshan | 24.53 | 101.02 | 2476 | Forest | 1848.33 | Tan et al. (2011), Yu et al. (2013) |
| 6 | Qianyanzhou | 26.73 | 115.05 | 100 | Forest | 1798.74 | Yu et al. (2013) |
| 7 | Huitong | 26.83 | 109.75 | 330 | Forest | 1524.95 | Zhang et al. (2010b), Zhao et al. (2011) |
| 8 | Ningxiang | 28.33 | 112.57 | 110 | Forest | 1963.6 | Jia et al. (2015) |
| 9 | Taoyuan | 28.92 | 111.45 | 92 | Cropland | 1598.54 | Zhu et al. (2005) |
| 10 | Yueyang | 29.53 | 112.86 |  | Wetland | 1974.80 | Han et al. (2008) |
| 11 | Damxung | 29.67 | 91.33 | 4250 | Grassland | 197.46 | Yu et al. (2013) |
| 12 | Tianmushan | 30.35 | 119.44 | 1139 | Forest | 1669.23 | Niu et al. (2016) |
| 13 | Anji | 30.47 | 119.67 | 380 | Forest | 1515 | Chen et al. (2016) |
| 14 | Xiping | 33.35 | 113.91 | 49 | Forest | 1288.10 | Geng et al. (2011) |
| 15 | Zoige | 33.93 | 102.87 | 3430 | Wetland | 630.95 | Hao et al. (2011) |
| 16 | Guoluo-graded | 34.35 | 100.55 | 3980 | Grassland | 464.4 | Wang et al. (2012a) |
| 17 | Guoluo | 34.35 | 100.55 | 3980 | Grassland | 511.9 | Wu et al. (2010), Wang et al. (2017) |
| 18 | Xiaolangdi | 35.02 | 112.47 | 410 | Forest | 1195.8 | Tong et al. (2010) |
| 19 | Changwu | 35.23 | 107.68 | 1220 | Cropland | 512.4 | Wang et al. (2013) |
| 20 | Weishan | 36.65 | 116.05 | 30 | Cropland | 1838 | Lei et al. (2010a, b) |
| 21 | Qinghai lake | 36.70 | 100.78 | 3214 | Wetland | 642.11 | Cao et al. (2017) |
| 22 | Yucheng | 36.83 | 116.57 | 28 | Cropland | 1746.62 | Yu et al. (2013) |
| 23 | Haibei | 37.62 | 101.30 | 3250 | Grassland | 634.50 | Kato et al. (2006) |
| 24 | Haibei-shrub | 37.67 | 101.33 | 3293 | Grassland | 511.61 | Li et al. (2016) |
| 25 | Haibei-wetland | 37.68 | 101.31 | 3160 | Wetland | 489.12 | Yu et al. (2013) |
| 26 | Yanchi | 37.71 | 107.23 | 1530 | Grassland | 357.67 | Jia et al. (2016) |
| 27 | Shouyang | 37.75 | 113.20 | 1202 | Cropland | 1340.67 | Gao et al. (2017) |
| 28 | Yellow river delta | 37.76 | 118.99 |  | Wetland | 809.67 | Han et al. (2015) |
| 29 | Arou | 38.03 | 100.45 | 3032 | Grassland | 853 | Wang et al. (2012b) |
| 30 | Daxing | 39.53 | 116.25 | 30 | Forest | 1452.56 | Zha et al. (2007), Fang et al. (2011) |
| 31 | KBQ | 40.38 | 108.55 | 1160 | Grassland | 270.18 | FLUXNET |
| 32 | Panjin | 41.15 | 121.92 | 7 | Wetland | 1298.16 | Zhou et al. (2009), Zhou et al. (2010) |
| 33 | Siziwang | 41.79 | 111.89 |  | Grassland | 107 | Shao et al. (2013) |
| 34 | Siziwang-graze | 41.79 | 111.89 |  | Grassland | 120.5 | Shao et al. (2013) |
| 35 | Duolun-Crop | 42.05 | 116.67 | 1350 | Cropland | 381 | Zhang et al. (2007), FLUXNET |
| 36 | Duolun-Grass | 42.05 | 116.28 | 1350 | Grassland | 370.69 | Zhang et al. (2007), FLUXNET |
| 37 | Changbaishan | 42.40 | 128.10 | 736 | Forest | 1338.84 | Yu et al. (2013) |
| 38 | Khorchin | 43.29 | 122.28 | 203 | Grassland | 1103.9 | Jing et al. (2014) |
| 39 | Xilinhot-fence | 43.55 | 116.67 | 1250 | Grassland | 294.00 | FLUXNET |
| 40 | Xilinhot-graded | 44.08 | 113.57 | 970 | Grassland | 149 | FLUXNET |
| 41 | Xilinhot | 44.13 | 116.33 | 1030 | Grassland | 130.02 | Wang et al. (2008a) |
| 42 | Fukang | 44.28 | 87.93 | 475 | Grassland | 151 | Ma et al. (2014) |
| 43 | Tongyu-Crop | 44.57 | 122.92 | 184 | Cropland | 283.28 | Du et al. (2013) |
| 44 | Tongyu-Grass | 44.59 | 122.52 | 184 | Grassland | 271.28 | Du et al. (2013) |
| 45 | Inner Mongolia | 44.53 | 116.67 | 1189 | Grassland | 231.66 | Yu et al. (2013) |
| 46 | Songneng | 44.58 | 123.50 | 171 | Grassland | 647.94 | Qu et al. (2016) |
| 47 | Laoshan | 45.33 | 127.67 | 370 | Forest | 1254.53 | Wang et al. (2008b), Cui et al. (2007) |
| 48 | Sanjiang | 47.58 | 133.52 | 55 | Wetland | 455.42 | Song et al. (2007) |
| 49 | Huzhong | 51.78 | 123.02 | 773 | Forest | 739.03 | Chen et al. (2013) |

**References**

Cao, S., Cao, G., Feng, Q., Han, G., Lin, Y., Yuan, J., Wu, F., Cheng, S., 2017. Alpine wetland ecosystem carbon sink and its controls at the Qinghai Lake. Environ. Earth Sci. 76: 210.

Chen, X.F., Jiang, H., Niu, X.D., Zhang, J.M., Liu, Y.L., Fang, C.Y., 2016. Effect of seasonal high temperature and drought on carbon flux of bamboo forest ecosystem in subtropical region. Chinese J. Appl. Ecol. 27(2): 335-344.

Chen, Z., Yu, G.R., Ge, J.P., et al. 2013. Temperature and precipitation control of the spatial variations of terrestrial ecosystem carbon exchange in the Asian region. Agr. Forest Meteorol. 182-183: 266-276.

Cui, S., 2007. Study in the CO2 flux of a Larch plantation in NE China by the micrometeorological method. Northeast Forestry University, Master Dissertation (in Chinese with an English abstract).

Du, Q., Liu, H.Z. 2013. Seven years of carbon dioxide exchange over a degraded grassland and a cropland with maize ecosystems in a semiarid area of China. Agric. Ecosyst. Envior. 173: 1-12.

Fang, X.R., 2011. Carbon exchange and its response to environmental factors in Poplar plantation ecosystem. Beijing Forestry University. Doctoral Dissertation (in Chinese with an English abstract).

Fei, X.H., Jin, Y., Zhang, Y.P., Sha, L.Q., Liu, Y., et al., 2017. Eddy covariance and biometric measurements show that a savanna ecosystem in Southwest China is a carbon sink. Sci. Rep. 7: 41025.

Gao, X., Gu, F., Hao, W., Mei, X., Li, H., Gong, D., Mao, L., Zhang, Z. 2017. Carbon budget of a rainfed spring maize cropland with straw returning on the Loess Plateau, China. Sci. Total Environ. 586: 1193-1203.

Geng, S.B., 2011. Study on the carbon flux observation over poplar plantation ecosystem of Xiping city in Hanan province of China. Beijing Forestry University. Master Dissertation (in Chinese with an English abstract).

Han, G., Chu, X., Xing, Q., Li, D., Yu, J., Luo, Y., Wang, G., Mao, P., Rafique, R. 2015. Effects of episodic flooding on the net ecosystem CO2 exchange of a supratidal wetland in the Yellow River Delta. Journal of Geophysical Research: Biogeosciences 120: 1506-1520.

Han, S., 2008. Productivity estimation of the poplar plantations on the beaches in middle and low reaches of Yangtze river using eddy covariance measurement. Chinese Academy of Forestry Doctoral Dissertation (in Chinese with an English abstract).

Hao, Y.B., Cui, X.Y., Wang, Y.F., Mei, X.R., Kang, X.M., Wu, N., Luo, P., Zhu, D. 2011. Predominance of Precipitation and Temperature Controls on Ecosystem CO_2_ Exchange in Zoige Alpine Wetlands of Southwest China. Wetlands 31: 413-422.

Jia, B., Xie, Z., Zeng, Y., Wang, L., Wang, Y., Xie, J., Xie, Z. 2015. Diurnal and seasonal variations of CO2 fluxes and their climate controlling factors for a subtropical forest in Ningxiang. Adv. Atmos. Sci. 32: 553-564.

Jia, X., Zha, T., Gong, J., Wang, B., Zhang, Y., Wu, B., Qin, S., Peltola, H. 2016. Carbon and water exchange over a temperate semi-arid shrubland during three years of contrasting precipitation and soil moisture patterns. Agric. For. Meteorol. 228-229: 120-129.

Jing, Y., Wang, A., Guan, D., Wu, J., Yuan, F., Jin, C. 2014. Carbon dioxide fluxes over a temperate meadow in eastern Inner Mongolia, China. Environ. Earth Sci. 72: 4401-4411.

Kato, T., Tang, Y., Gu, S., Hirota, M., Du, M., Li, Y., Zhao, X., 2006. Temperature and biomass influences on interannual changes in CO2 exchange in an alpine meadow on the Qinghai-Tibetan Plateau. Global Change Biol. 12: 1285-1298.

Lei, H.M., Yang, D.W., 2010a. Seasonal and interannual variations in carbon dioxide exchange over a cropland in the North China Plain. Global Change Biol. 16(11): 2944-2957.

Lei, H.M, Yang, D.W, 2010b. Interannual and seasonal variability in evapotranspiration and energy partitioning over an irrigated cropland in the North China Plain. Agr. Forest. Meteorol. 150(4): 581-589.

Li, H., Zhang, F., Li, Y., Wang, J., Zhang, L., Zhao, L., Cao, G., Zhao, X., Du, M. 2016. Seasonal and inter-annual variations in CO2 fluxes over 10 years in an alpine shrubland on the Qinghai-Tibetan Plateau, China. Agric. For. Meteorol. 228-229: 95-103.

Ma, J., Liu, R., Tang, L.S., Lan, Z.D., Li, Y. 2014. A downward CO_2_ flux seems to have nowhere to go. Biogeosciences 11: 6251-6262.

Niu, X.D., Jiang, H., Zhang, J.M., Fang, C.Y., Chen, X.F., Sun, H., 2016. Characteristics of CO_2_ flux in an old growth mixed forest in Tianmu Mountain, Zhejiang, China. Chinese J. Appl. Ecol. 27(1): 1-8.

Qu, L., Chen, J., Dong, G., Jiang, S., Li, L., Guo, J., Shao, C. 2016. Heat waves reduce ecosystem carbon sink strength in a Eurasian meadow steppe. Environ. Res. 144: 39-48.

Shao, C.L.,Chen, J.Q., Li, L.H. 2013. Grazing alters the biophysical regulation of carbon fluxes in a desert steppe. Environ. Res. Lett. 8: 025012.

Song, T., 2007. Long term carbon dioxide flux measurements in Sanjiang plain, Northeastern China. Nanjing University of Information Science and Technology Doctoral Dissertation (in Chinese with an English abstract).

Sun, C.J., 2012. Study of CO_2_ flux above urban green space in Peal River Delta. Nanjing University of Information Science and Technology. Master Dissertation (in Chinese with an English abstract).

Tan, Z.H., Zhang, Y.P., Schaefer, D., Yu, G.R., Liang, N., Song, Q.H., 2011. An old-growth subtropical Asian evergreen forest as a large carbon sink. Atmos. Environ. 45(8): 1548-1554.

Tong, X.J., Zhang, J.S., Meng, P., Huang, H., Guo, L., Yin, C.J., Gao, J., 2010. Characteristics of net carbon exchange over a mixed plantation in a hilly area of the north China. Scientia Silvae Sinicae 46(3): 37-43 (in Chinese with an English abstract).

Wang, B., Li, J., Jiang, W.W., Zhao, L., Gu, S., 2012a. Impacts of the rangeland degradation on CO_2_ flux and the underlying mechanisms in the Three-River Source Region on the Qinghai-Tibetan Plateau. China Envir. Sci. 32(10): 1764-1771.

Wang, X., Ma, M., Huang, G., Veroustraete, F., Zhang, Z., Song, Y., Tan, J. 2012b. Vegetation primary production estimation at maize and alpine meadow over the Heihe River Basin, China. Int. J. Appl. Earth Obs. Geoinf. 17: 94-101.

Wang, B., Jin, H.Y., Li, Q., Chen, D.D.,Zhao, L.,Tang, Y.H., Kato, T., Gu, S. 2017. Diurnal and seasonal variations in the net ecosystem CO_2_ exchange of a pasture in the Three-River source region of the Qinghai−Tibetan Plateau. PLoS ONE 12(1): e0170963.

Wang, Y.L, Zhou, G.S, Wang, Y.H, 2008a. Environmental effects on net ecosystem CO_2_ exchange at half-hour and month scales over Stipa krylovii steppe in northern China. Agr. Forest. Meteorol. 148(5): 714-722.

Wang, H.M., Saigusa, N., Zu, Y.G., Wang, W.J., Yamamoto, S., Kondo, H., 2008b. Carbon fluxes and their response to environmental variables in a Dahurian larch forest ecosystem in northeast China. J. For. Res. 19(1): 1-10.

Wang, W., Liao, Y., Wen, X., Guo, Q. 2013. Dynamics of CO_2_ fluxes and environmental responses in the rain-fed winter wheat ecosystem of the Loess Plateau, China. Sci. Total Environ. 461-462: 10-18.

Wu, L.B., Gu, S., Zhao, L., Xu, S.X., Zhou, H.K., Feng, C., Xu, W.X., Li, Y.N., Zhao, X.Q., Tang, Y.H., 2010. Variation in net CO_2_ exchange, gross primary production and its affecting factors in the planted pasture ecosystem in Sanjiangyuan Region of the Qinghai-Tibetan Plateau of China. Chinese J. Plant Ecol. 34(7): 770–780.

Yu, G.R., Zhu, X.J., Fu, Y.L., et al. 2013. Spatial patterns and climate drivers of carbon fluxes in terrestrial ecosystems of China. Global Change Biol. 19: 798-810.

Zha, T.G., 2007. Carbon balance of a poplar plantation ecosystem in Daxing, Beijing. Beijing Forestry University. Doctoral Dissertation (in Chinese with an English abstract).

Zhao, Z.H., 2011. A study on carbon flux between Chinese Fir plantation and atmosphere in Subtropical Belts. Central South University of Forestry and Technology, Doctoral Dissertation (in Chinese with an English abstract).

Zhang, W.L., Chen, S.P., Chen, J., Wei, L., Han, X.G., Lin, G.H., 2007. Biophysical regulations of carbon fluxes of a steppe and a cultivated cropland in semiarid Inner Mongolia. Agr. Forest. Meteorol. 146(3-4): 216-229.

Zhang, Y., Tan, Z., Song, Q., Yu, G., Sun, X., 2010a. Respiration controls the unexpected seasonal pattern of carbon flux in an Asian tropical rain forest. Atmos. Environ., 44(32): 3886-3893.

Zhang, L.P., 2010b. Characteristics of CO_2_ flux in a Chinese Fir plantation ecosystem in Huitong County, Hunan Province. Central South University of Forestry and Technology, Master Dissertation (in Chinese with an English abstract).

Zhou, L., Zhou, G., Jia, Q., 2009. Annual cycle of CO_2_ exchange over a reed (Phragmites australis) wetland in Northeast China. Aquat. Bot. 91(2): 91-98.

Zhou, L., Zhou, G., Liu, S., Sui, X., 2010. Seasonal contribution and interannual variation of evapotranspiration over a reed marsh (Phragmites australis) in Northeast China from 3-year eddy covariance data. Hydrol. Processes 24(8): 1039-1047.

Zhu, Y.L., 2005. Carbon dioxide exchange between Paddy ecosystem and the atmosphere in the Subtropical region. Chinese Academy of Sciences, Doctoral Dissertation (in Chinese with an English abstract).
